# A snapshot on HIV-1 evolution through the identification of phylogenetic-specific properties of HIV-1 integrases M/O

**DOI:** 10.1371/journal.ppat.1011207

**Published:** 2023-03-30

**Authors:** Elenia Toccafondi, Marine Kanja, Flore Winter, Daniela Lener, Matteo Negroni

**Affiliations:** 1 Architecture et Réactivité de l’ARN-UPR 9002, IBMC, CNRS, Université de Strasbourg, Strasbourg, France; 2 Interdisciplinary Thematic Institute (ITI) InnoVec, Université de Strasbourg, Strasbourg, France; Boston College, UNITED STATES

## Abstract

Transmissions of simian viruses to humans has originated the different groups of HIV-1. We recently identified a functional motif (CLA), in the C-terminal domain of the integrase, essential for integration in HIV-1 group M. Here, we found that the motif is instead dispensable in group O isolates, because of the presence, in the N-terminal domain of HIV-1 O of a specific sequence, Q_7_G_27_P_41_H_44_, that we define as the NOG motif. Alterations of reverse transcription and of 3’ processing observed by mutating the CLA motif of IN M are fully rescued to wt levels by inserting the sequence of the NOG motif in the N-ter of the protein. These results indicate that the two motifs (CLA and NOG) functionally complement each other and a working model accounting for these observations is proposed. The establishment of these two alternative motifs seems to be due to the different phylogenetic origin and history of these two groups. Indeed, the NOG motif is already present in the ancestor of group O (SIVgor) while it is absent from SIVcpz*Ptt*, the ancestor of group M. The CLA motif, instead, seems to have emerged after SIVcpz*Ptt* has been transferred to humans, since no conservation is found at the same positions in these simian viruses. These results show the existence of two-group specific motifs in HIV-1 M and O integrases. In each group, only one of the motifs is functional, potentially leading the other motif to diverge from its original function and, in an evolutionary perspective, assist other functions of the protein, further increasing HIV genetic diversity.

## Author summary

HIV-1 establishes a permanent infection by integrating its genome in the one of the host cell. This key step is achieved by the viral integrase (IN), which is composed of three domains: N-terminal (NTD), catalytic core (CCD), and C-terminal (CTD). We recently reported, in the CTD, the existence of a functional motif (CLA) that is essential for integration in HIV-1 group M. Here we show that in HIV-1 group O, the function exerted by the integrase M CLA motif is instead ensured by another motif (NOG), located in the NTD of the protein. We show that these two motifs can functionally complement each other by acting on the same steps of the viral cycle, highlighting for the first time such an important functional duality between HIV-1 integrases. We also show that, while the NOG motif in HIV-1 O was likely inherited from its simian ancestor (SIVgor), the CLA motif seems to have emerged in the human host, probably in the process of adaptation to the new host of the simian virus on its way to become HIV-1 M. Such a requirement did not seem to have existed for HIV-1 O, possibly because of the presence of the NOG motif.

## Introduction

Transmission of viruses from animals to human is a main threat to human health, with the HIV-1 pandemic being a clear example of this. The four HIV-1 groups, in fact, all originated from an independent zoonotic transmission of simian viruses to humans. Group M and group N both derive from SIVcpz*Ptt* [1,2], while group O and P derive from SIVgor [3,4]. Although HIV-1 groups M and O share similar geographic and temporal origins [5–7], they encountered a largely different epidemiological success. While group M is the responsible for the AIDS pandemic, infecting around 39 million people all over the world, group O has a largely lower epidemiological success, infecting around 30 thousand people mostly in the west-central region of Africa [8,9]. The bases for this discrepancy are only partially known to date, although they constitute a central question to identify critical properties allowing cross-species transmission and diffusion. Their different zoonotic origin and the subsequent sequence diversification in the human host are responsible for the large intergroup genetic diversity between groups M and O that can reach almost 50% in the *env* gene [10]. Despite this, they have globally convergent phenotypes and, to date, only few functional differences have been highlighted between their proteins and enzymes. Among those, the most marked one concerns the counteraction of the antiviral properties of the cellular protein tetherin, that is exerted by Vpu in HIV-1 M while it is partially carried out by Nef in the case of HIV-1 O [11–14].

HIV replication requires the integration of the reverse transcribed genomic RNA into the genome of the infected cell. This key step is catalyzed by the integrase (IN), one of three viral enzymes. Integrases M and O share 84% of sequence identity as well as the same domain organizations and the same functions. IN is constituted by three domains connected by flexible linkers: the N-terminal domain (NTD), the catalytic core domain (CCD), and the C-terminal domain (CTD) [15–17]. Each of these domains is specialized in one or more functions. The NTD is important for the multimerization and stabilization of the active form of the integrase [18,19], which is a highly organized multimer formed by several dimers of dimers [20,21]. The CCD is involved in DNA binding and contains the amino acidic triad responsible for the catalytic activity of the enzyme [22], but it is also the domain involved in protein dimerization and it is in charge of the interaction with LEDGF/p75, a fundamental host factor required for the successful infection by HIV-1 [23]. Finally, the CTD is involved in binding viral RNA/DNA at different steps of the infectious cycle [24–27], and in the interaction with the viral reverse transcriptase [28,29].

It is in the CTD of the IN from group M that we previously identified a functional motif, constituted by four non-contiguous amino acids (positions 222, 240, 254, and 273) [30]. We will refer to the sequence N_222_K_240_N_254_K_273_ of integrases M (that is the one yielding the highest levels of integration in group M while also assuring the highest levels of reverse transcription) as the "CLA (C-terminal lysine-amidic) motif" and to the same positions, irrespectively of the amino acids harbored, as the CLA positions. Despite its high conservation *in vivo*, the positions of the four residues could be permutated within the motif, in most cases, without affecting the efficiency of integration in cell culture [30]. In fact, as long as at least two lysine are present within the motif and the remainders are amidic residues (N or Q), functionality is retained to wt levels in most of the possible combinations [30]. The combination where the integration efficiency was the most affected, dropping to 25% of the wt, is NQKK. Also, other steps of the viral cycles are affected when this sequence was inserted in HIV-1 M IN. Indeed, a drop in the amount of the reverse transcription products and in the amount of the 3’ processed ends was observed [30]. We previously determined the structure of the CTD for this variant (NQKK) showing that the protein folded into a structure similar to that of the wt CTD (N_222_K_240_N_254_K_273_), but with a different distribution of charges at its surface [30], which could participate in the observed impairment of functionality. In the wt conditions, indeed, the CLA motif forms a positive surface that was proposed to bind to a negatively charged partner, while, when the amino acidic sequence NQKK is inserted at the CLA positions, the positive surface is lost [30]. Astoundingly, we found that this aminoacidic sequence (N_222_Q_240_K_254_K_273_) is highly conserved in group O CLA positions, raising the question of how could have been selected in group O a motif with such a markedly reduced functionality compared to the motif of group M. Understanding whether a possible functional difference between the integrases of HIV-1 isolates M and O might exist could help us understanding the reasons behind their different success. Therefore, investigating the role of the CLA motif functionality in the IN of HIV-1 O isolates constituted the starting point of this work.

## Results

### The CLA motif is dispensable in isolates of group O

**Fig 1:**
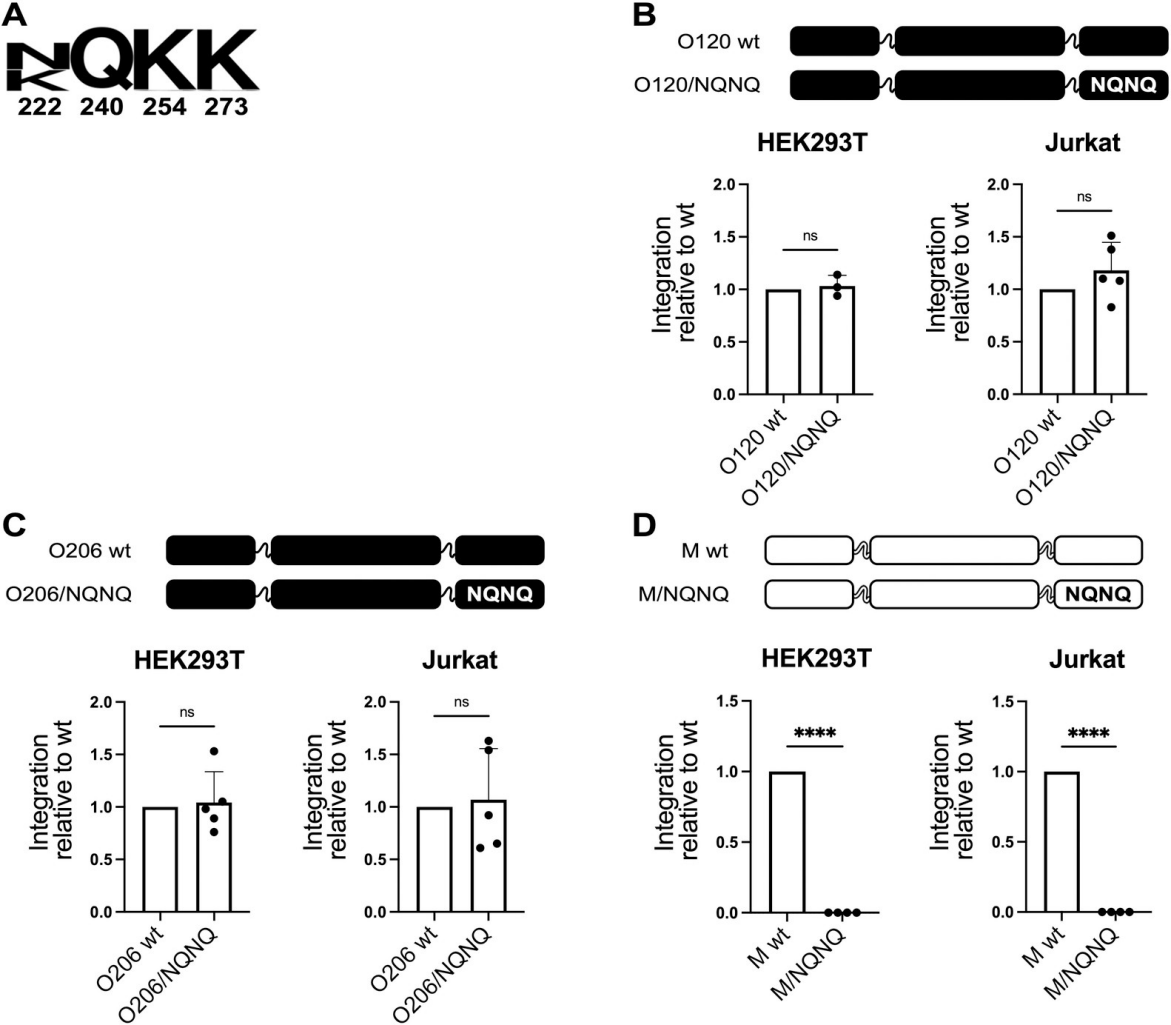
The CLA motif is dispensable in isolates of group O. (**A**) Sequence conservation logo of the CLA motif positions in isolates of group O. (**B**-**D**) Top of each panel: schematic representation of IN tested for integration. Color code is white for isolates M and black for isolates O. When mutated with respect to the sequence of the wt, the amino acids of the CLA motif are shown in capital letters. Bottom of each panel: normalized levels of integration relative to the level of the wt IN. (**B**) n = 3 for HEK293T and n = 5 for Jurkat. (**C**) n = 5. (**D**) n = 4. Data are shown as the average ± SD. ****p ≤ 0.0001. ns, not significant (two-tailed, unpaired Student’s t-test).

While, as mentioned above, the influence on integration of the amino acids that occupy the CLA positions has been well characterized for HIV-1 group M, their effect is unknown for group O isolates. To shed light on this aspect, we used two isolates from this group, BCF120 and RBF206 (named hereafter O120 and O206, respectively) that present in the CLA positions either the same sequence as the group O consensus sequence (NQKK, isolate O120, Fig 1A) or a different one (KQKQ, isolate O206 that was chosen as outlier). In both isolates we replaced the sequence in the CLA positions by NQNQ (O120/NQNQ and O206/NQNQ, Fig 1B and 1C), a sequence that was shown to abolish integration in isolates M [30]. In sharp contrast to what observed for group M, for both isolates the replacement of the original sequence in the CLA positions by NQNQ did not affect integration neither in HEK293T nor in Jurkat cells (Fig 1B and 1C). The same replacement in isolate M AF286237 (referred hereafter as "isolate M"), used as a control, led to undetectable levels of integration (Fig 1D). These observations indicate that either isolates O do not require the function exerted by the CLA motif or that this function is endorsed by another region of the integrase or by another protein.

### The NTD of isolates O complements the function of the CLA motif of isolates M

**Fig 2:**
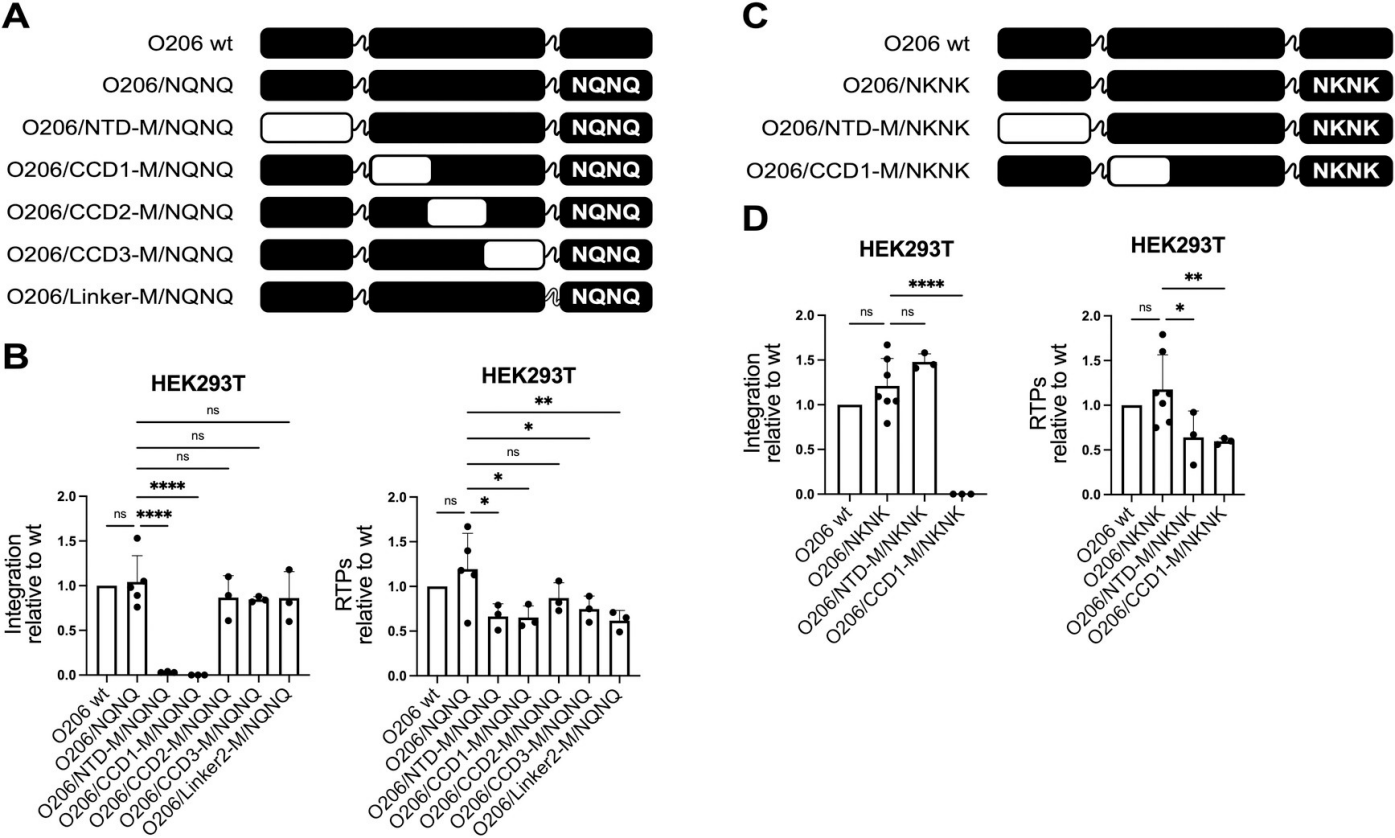
The NTD of isolates O complements the function of the CLA motif of isolate M. (**A**) Schematic representation of the chimeras with the NQNQ sequence in the CLA motif positions and of IN O wt, as reference at the top of the drawing. Color code is black for isolates O and white for isolates M. (**B**) Normalized levels of integration (left graph) and amount of RTPs (right graph), relative to the wt IN, for the chimeras shown in panel A (n = 5 for O206 wt and O206/NQNQ, n = 3 for the remaining samples). (**C**) Schematic representation of the mutants used to discern whether the loss of functionality of the two chimeras shown in panel B is related to the functionality of the CLA motif. (**D**) Normalized levels of integration (left graph) and amount of RTPs (right graph), relative to the wt IN, for the chimeras shown in panel C (n = 7 for O206 wt and O206/NKNK, n = 3 for the remaining samples). Data are shown as the average ± SD. ****p ≤ 0.0001. **p ≤ 0.01. *p ≤ 0.05. ns, not significant (one-way ANOVA with Tukey’s multiple comparisons correction).

We first investigated whether another region of group O integrases exerts the same function of the integrases M CLA motif. To this end, we replaced, in O206/NQNQ, five large regions with the homologous ones of isolate M and measured integration in HEK293T (Fig 2A). In isolate M, the replacement of the NKNK sequence, the CLA motif, by NQNQ was sufficient to abolish integration (Fig 1B) indicating that no other region complements the default in the CLA motif in this group. Therefore, if the region that ensures the functions of the CLA motif in O206/NQNQ is replaced by the homologous region of isolate M, integration should no longer occur.

Integrase is a pleiotropic protein. As such, if mutated, it can influence different steps of the infectious cycle, several of which can affect the generation of proviral DNA. Among these are reverse transcription and, when IN is still part of the Gag-Pol precursor, Pr160Gag-Pol proteolytic processing, a step required to obtain a mature infectious particle. The decrease in the number of provirus generated with a mutated IN could therefore be due either to a default in integration *per se*, or to a default in the steps preceding integration. For these reasons, for each mutant generated in this work, we evaluated, besides the formation of proviral DNA, the efficiency of reverse transcription and that of Pr55Gag proteolytic processing. Furthermore, if less reverse transcription products (RTPs) are produced with a mutant, less proviral DNAs will be generated even if the mutant is not affected in the step of integration itself. For this reason, to measure the efficiency of integration *per se* we expressed the levels of integration normalized by the amount of late reverse transcription products throughout the study (see Methods).

The estimates of the efficiency of integration for the chimeras shown in Fig 2A clearly indicate that integration was abolished for two of them (chimeras O206/NTD-M/NQNQ and O206/CCD1-M/NQNQ, Fig 2B), corresponding to the chimeras where either the NTD or the N-terminal part of the CCD were replaced by the homologous regions of isolate M. The effect on these two mutants was specific for integration since proteolytic processing of Pr55Gag was unaffected with respect to wt IN O206 in all chimeras as well as in O206/NQNQ (S1A and S1B Fig) while reverse transcription was reduced to approximately 60% of wt IN O206, although in a comparable manner across the chimeras (Fig 2B).

The inability of O206/NTD-M/NQNQ and O206/CCD1-M/NQNQ to produce proviral DNA could be due to the absence of the functionality provided by the equivalent of the CLA motif or to other defects such as, for example, protein misfolding. To ascertain whether the lack of integration was related to the absence of the region that ensures the function of the CLA motif, we replaced NQNQ (non-functional CLA motif) by NKNK (functional CLA motif), obtaining chimeras O206/NTD-M/NKNK and O206/CCD1-M/NKNK (Fig 2C). We also inserted the sequence NKNK in wt IN O206 (O206/NKNK) to verify that this insertion did not affect the functionality of the enzyme. As shown in Fig 2D, neither integration nor reverse transcription were affected in this mutant. Integration was fully restored for the chimera containing the NTD M, while it remained undetectable for O206/CCD1-M/NKNK (Fig 2D). Therefore, the default of chimera O206/NTD-M/NQNQ appears related to the lack of the region that exerts the function of the CLA motif, whereas for O206/CCD1-M/NKNK the loss of integration was unrelated to the functionality ensured by the CLA motif (Figs 2D and S1B). These results indicate that the NTD of isolate O206 can complement the absence of a functional CLA motif. Furthermore, the high similarity between the NTD of O206 and the consensus sequence O (only one substitution, K46R; S2 Fig), suggests that this is likely the case for integrases of HIV-1 group O in general.

### Identification and characterization of the N-terminal O group (NOG) motif

**Fig 3:**
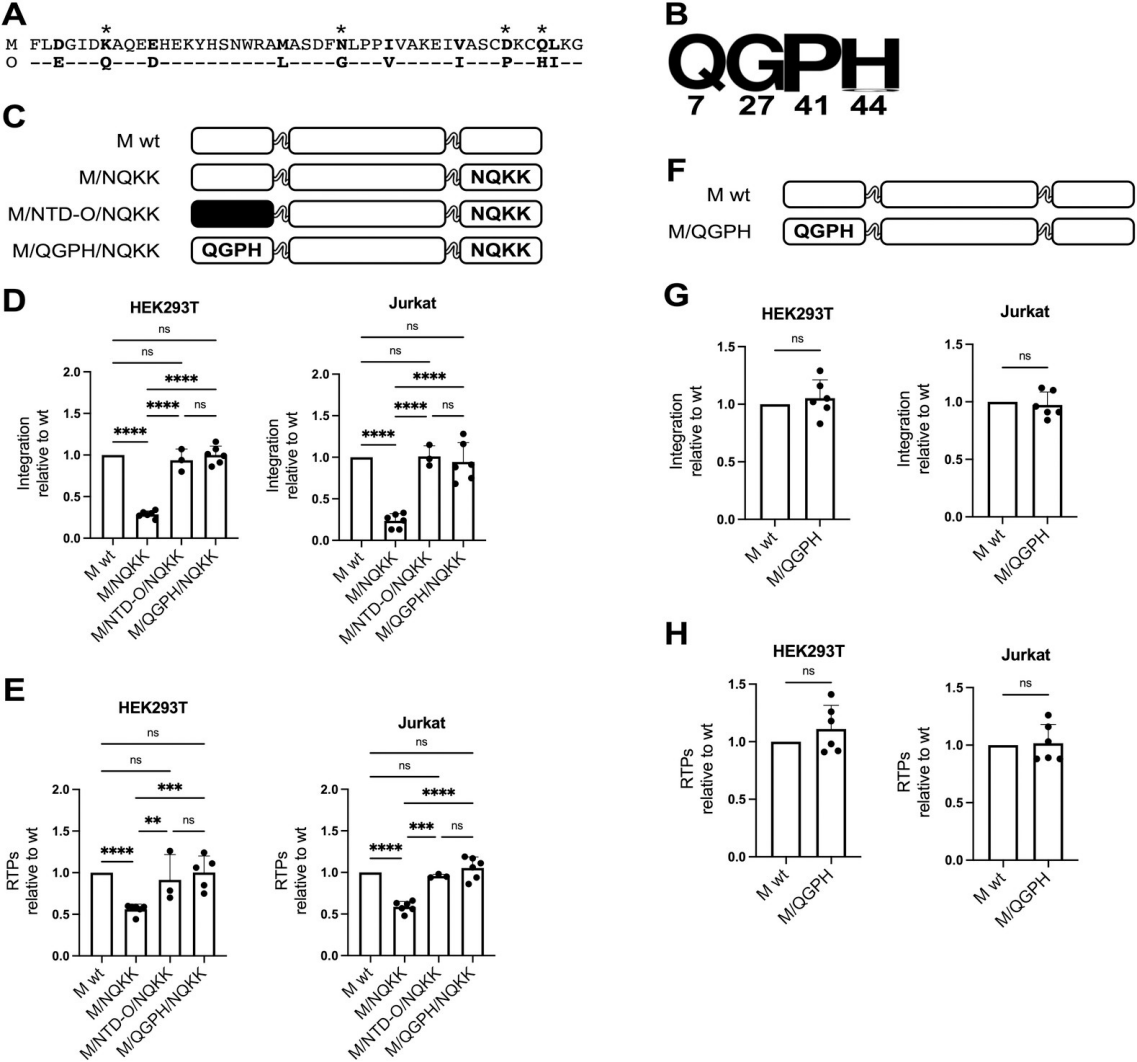
Identification and characterization of the N-terminal O group (NOG) motif. (**A**) Alignment of the amino acid sequences of the NTD of IN M (top row) and IN O (bottom row). Unchanged amino acids in IN O with respect to IN M are indicated by a dash. Positions differing in the two sequences are in bold. Residues whose replacement gives a BLOSUM62 matrix score difference ≤ 1 are highlighted by a star. (**B**) Sequence conservation logo for positions 7, 14, 41 and 44 of IN O. (**C**) Schematic representation of the mutant IN used to evaluate the function of the NOG motif. White for isolate M and black for isolate O120. When mutated with respect to the sequence of the wt, the amino acids of the NOG or of the CLA motifs are shown in capital letters. (**D**) Normalized levels of integration relative to the wt IN, for the chimeras shown in panel C (n = 3 for M/NTD-O/NQKK; n = 6 for all the remaining samples). (**E**) Amounts of RTPs, relative to the wt IN, for the chimeras shown in panel C (n = 3 for M/NTD-O/NQKK; n = 6 for all the remaining samples). (**F**) Schematic representation of IN M/QGPH. (**G** and **H**) Normalized levels of integration (panel G) and amount of RTPs (panel H), relative to the wt IN, for IN M/QGPH (n = 6 for all the samples). Data are shown as the average ± SD. ****p ≤ 0.0001. ***p ≤ 0.001. **p ≤ 0.01. ns, not significant (one-way ANOVA with Tukey’s multiple comparisons correction for panels D and E. Two-tailed, unpaired Student’s t-test for panels G and H).

The consensus sequences of the NTDs M and O differ for 10 residues (Fig 3A). According to the score of the BLOSUM62 matrix [31], the replacement of four of these residues (Q7, G27, P41, H44, highlighted by a star in Fig 3A and highly conserved in group O as shown in Fig 3B) introduces more drastic changes in the properties of the protein than the substitution of the other residues.

To test if the four residues Q_7_G_27_P_41_H_44_ of the NTD O are the ones allowing for the complementation of the functionality ensured by the CLA motif, we inserted them in the NTD of the IN HIV-1 M that harbors, in the CLA positions, the same sequence as the consensus one of isolates O (IN M/QGPH/NQKK, Fig 3C). This double mutant recovered an integration efficiency from 25% of IN M/NQKK to 100% of wt IN M, both in HEK293T and Jurkat cells (Fig 3D). The same results were obtained by replacing the whole NTD M by the NTD O (IN M/NTD-O/NQKK in Fig 3D). For both cell types, the replacement of the QGPH, also led to an improvement of reverse transcription levels to the ones observed for the wt enzyme (Fig 3E). The four amino acids Q_7_G_27_P_41_H_44_, located in the NTD of HIV-1 O IN, are therefore sufficient to complement the decreases in integration and in reverse transcription that were generated by the replacement of the sequence NKNK by NQKK in the CLA motif of IN M. We will refer to them hereafter as the NOG (for “N-terminal O group”) motif. Finally, no differences were observed in the efficiency of Pr55Gag processing for all constructions compared to the wt (S1C Fig).

### Tracing the phylogenetic origins of the NOG and CLA motifs

**Fig 4:**
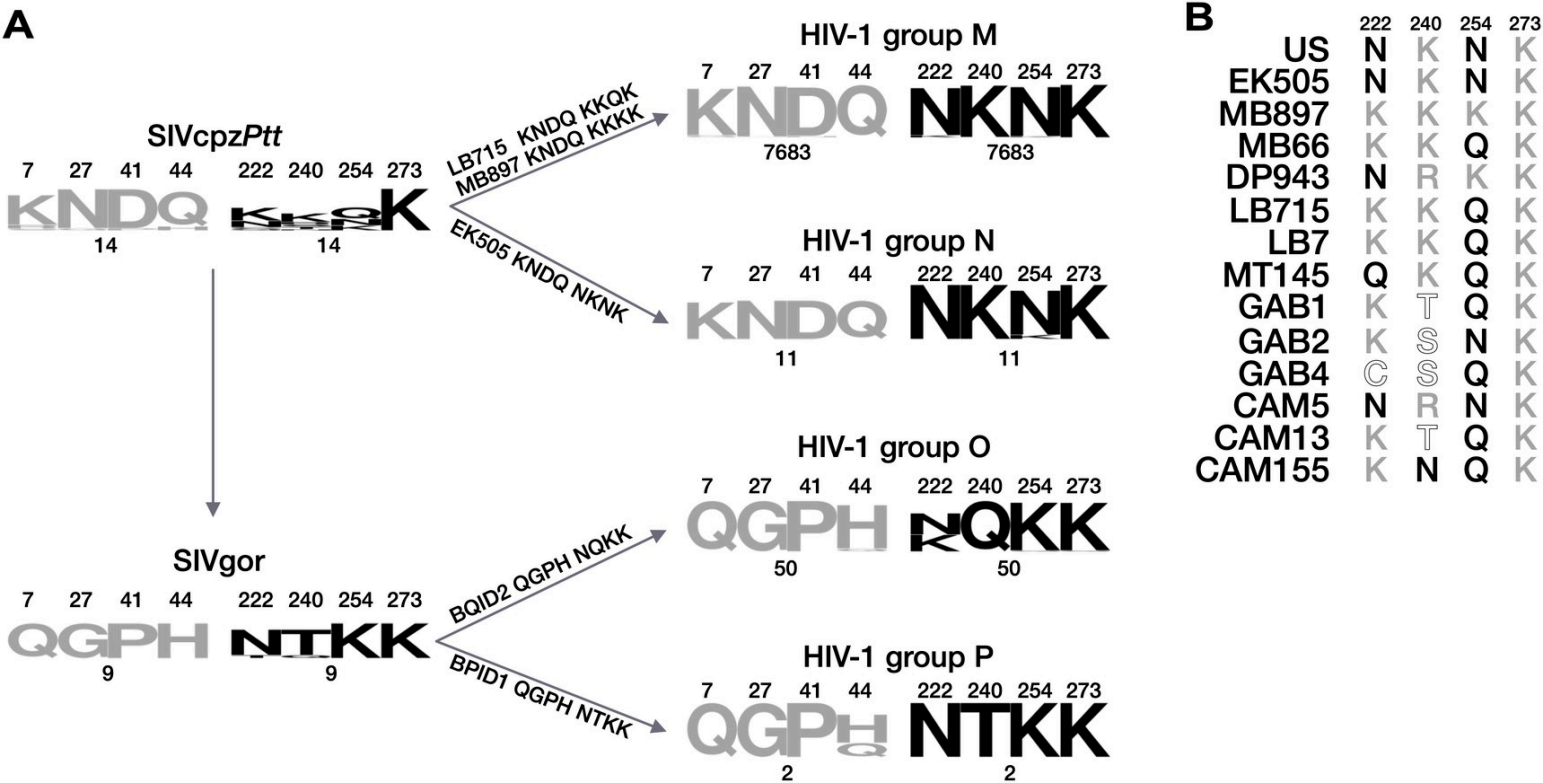
Tracing the phylogenetic origins of the NOG and CLA motifs. (**A**) Sequence conservation logos of the NOG (in grey) and CLA (in black) motifs are shown for HIV-1 groups (M, N, O, P) and their ancestor viruses (SIVgor and SIVcpz*Ptt*). The numbers above each logo indicate the amino acidic position in the IN. The number below each logo indicate the number of sequences that were aligned to obtain the logo. Each arrow represents a zoonotic transmission event. On or under each arrow are shown the name and the NOG and CLA sequences of the isolates phylogenetically most related to the HIV-1 group indicated by the arrow itself. (**B**) The CLA positions amino acidic sequence is shown for each SIVcpz*Ptt* isolate, which name are indicated on the left. Amidic amino acids are shown in black; basic amino acids are shown in grey; any other amino acid is shown in white (with a black outline).

To understand how these functional differences between IN from HIV-1 groups M and O could have emerged, we analyzed the NOG and the CLA positions in the simian viruses assumed to be the ancestors of these groups of HIV-1, SIVcpz*Ptt* and SIVgor, respectively (Fig 4A). Concerning the NOG motif, the sequence QGPH, is highly conserved in HIV-1 group O (see S3 Fig) and in SIVgor (Fig 4A) and it is also found in the isolate supposed to be the closest to the founder of HIV-1 O, SIVgor BQID2 [3] (Fig 4A). These observations suggest that this motif was inherited by HIV-1 O from its ancestor and that it has remained unaltered ever since. The possibility of a cross-species transmission event of the NOG motif from SIVgor to human viruses is further supported by the observation that also the other group of HIV-1 that originated from SIVgor (HIV-1 group P) carries the NOG motif in one of the two isolates identified so far (Fig 4A). The next question was then to understand whether the NOG motif emerged in SIVgor or if it was inherited from its ancestor. Indeed, SIVgor originated from a cross-species transmission event of SIVcpz*Ptt* in which, the NOG positions are occupied by the highly conserved sequence KNDQ (Fig 4A). The unrelatedness of the sequences found in the NOG positions in these two viruses, supports the view that the NOG motif emerged and was fixed in SIVgor. The amino acidic sequence found at the NOG positions of SIVcpz*Ptt* appears to be instead conserved in HIV-1 groups M and N, in which the same sequence (KNDQ) is found (Fig 4A).

The CLA motif seems to have been established in HIV-1 M, after SIVcpz*Ptt* had been transmitted to humans. Indeed, in SIVcpz*Ptt* no conservation is observed in the CLA positions, except for K_273_ (Fig 4A). Accordingly, none of the two isolates of SIVcpz*Ptt* considered to be the closest to HIV-1 M, SIVcpz*Ptt* MB897 and SIVcpz*Ptt* LB715 [2,32], carries the sequence NKNK (Fig 4A). Strikingly, when we looked at the conservation of the CTD region (200–280) of SIVcpz*Ptt* we found that it is overall conserved (S4 Fig). In fact, together with the first three CLA positions (222, 240, 254), only a few more show to have a conservation level of around 50% (211, 212, 220, 255, 278, 279, 280) (S4 Fig). Furthermore, no amidic or basic amino acids are exclusively found in SIVcpz*Ptt* IN CTD, in comparison to HIV-1 M IN CTD (S4 Fig), suggesting that no other positions could compensate for the CLA functionality in SIVcpz*Ptt* IN CTD. Despite the lack of conservation at SIVcpz*Ptt* CLA positions, though, a trend for the preference of basic (K, R) and amidic (N, Q) amino acids is observable (Fig 4B). At the level of the individual isolates, two SIVcpz*Ptt* (EK505 and US) have the NKNK sequence (Fig 4B). EK505 is the isolate most related to HIV-1 group N [2]. In this group, as in HIV-1 M, the sequence NKNK is highly conserved suggesting that transmission of this specific isolate, or isolates closely related to it, could be responsible for the presence of the motif in HIV-1 group N. Irrespectively of the path followed, it is worth to underline that the sequence NKNK was ultimately fixed in both groups of human viruses derived from SIVcpz*Ptt*. Concerning the relationship between HIV-1 O and SIVgor for the amino acids present in the CLA positions, the simian virus carries the conserved sequence NTKK that, in HIV-1 O, presents the replacement of T240 by a Q (NQKK, Fig 4A). This replacement could reflect adaptation to the new host, but it could also have been inherited from an isolate of SIVgor that had a Q at position 240, as for BQID2, that is furthermore assumed to be the closest to HIV-1 O (Fig 4A).

**Fig 5:**
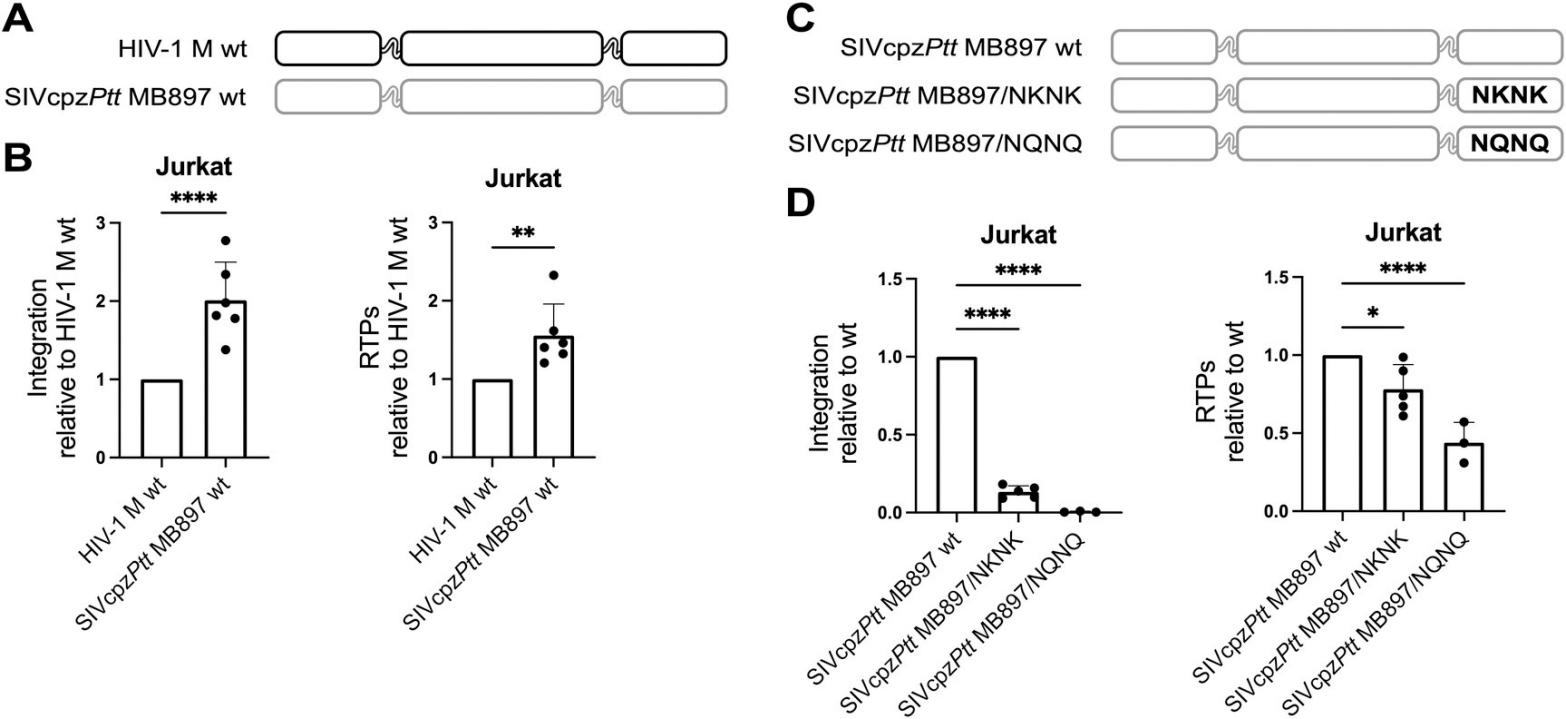
**SIVcpz*Ptt* CLA motif is important for integration** (**A**) Schematic representation of wt IN M and IN SIVcpz*Ptt* MB897. (**B**) Normalized levels of integration (left graph) and amounts of RTPs (right graph) relative to isolate M for the IN shown in panel A (n = 6). (**C**) Schematic representation of IN SIVcpz*Ptt* MB897 wt and the two mutants for the CLA motif positions tested for integration and reverse transcription in panel D. When mutated with respect to the sequence of the wt, the amino acids of the NOG or of the CLA motifs are shown in capital letters. (**D**) Normalized levels of integration (left graph) and amounts of RTPs (right graph) relative to SIVcpz*Ptt* MB897 wt (n = 6 for MB897 wt; n = 5 for SIVcpz*Ptt* MB897/NKNK; n = 3 for SIVcpz*Ptt* MB897/NQNQ). Data are shown as the average ± SD. ****p ≤ 0.0001. **p ≤ 0.01. *p ≤ 0.05. (Two-tailed, unpaired Student’s t-test for panel B. One-way ANOVA with Tukey’s multiple comparisons correction for panel D).

Once established that the sequence of the CLA motif (NKNK) of HIV-1 group M, was probably not inherited from SIVcpz*Ptt*, we wanted to understand if a virus with the IN from the closest to HIV-1 M SIVcpz*Ptt* MB897, but already endowed with the NKNK sequence in the CLA positions, could have been infectious in human cells. To address this issue, we first produced viral particles in which we replaced the RT and IN of HIV-1 M by those of isolate SIVcpz*Ptt* MB897, that has the KKKK sequence in the CLA positions (Fig 5A). The goal was to verify that the chimeric nature of these viruses (Gag and protease from HIV-1 M; RT and IN from SIV) was not an obstacle for infection. The chimeric particles were fully processed by the protease (S5A Fig) and integration levels were twice those obtained with wt IN M (Fig 5B). Reverse transcription was also increased with respect to wt IN M (Fig 5B). In conclusion, the chimeric nature of the virus, did not negatively affect its functionality, which, instead, was enhanced. These results are in line with other reports where it has been shown that SIVcpz*Ptt* isolate MB897 efficiently infects human cells, with kinetics more similar to HIV-1 M rather than other SIVcpz*Ptt* [33,34]. We then replaced the KKKK sequence in the CLA positions of the SIVcpz*Ptt* integrase by NKNK (Fig 5C). This change reduced integration to around 10% with respect to wt IN SIVcpz*Ptt* MB897 (Fig 5D) and, consequently, to around 20% of wt IN M. Reverse transcription and Pr55Gag processing were either only slightly (Fig 5D) or not at all altered (S5B Fig), respectively. This result markedly differs from what had been observed for IN M, for which the two sequences (KKKK and NKNK) yielded comparable levels of integration [30]. The replacement of the amino acids in the CLA positions by NQNQ, condition that abolished integration in IN M, caused a drop of integration to undetectable levels, as well as a significant decrease in reverse transcription levels (Fig 5D). Pr55Gag processing levels, instead, were still unaltered (S5B Fig). Altogether, these results indicate that in SIVcpz*Ptt*, the sequence in the CLA positions is crucial to determine the levels of integration, like for IN M, but in sharp contrast with IN M, the sequence NKNK was poorly functional, at least in the background of strain MB897 of SIVcpz*Ptt*.

### Functional complementarity of the NOG and CLA motifs

**Fig 6:**
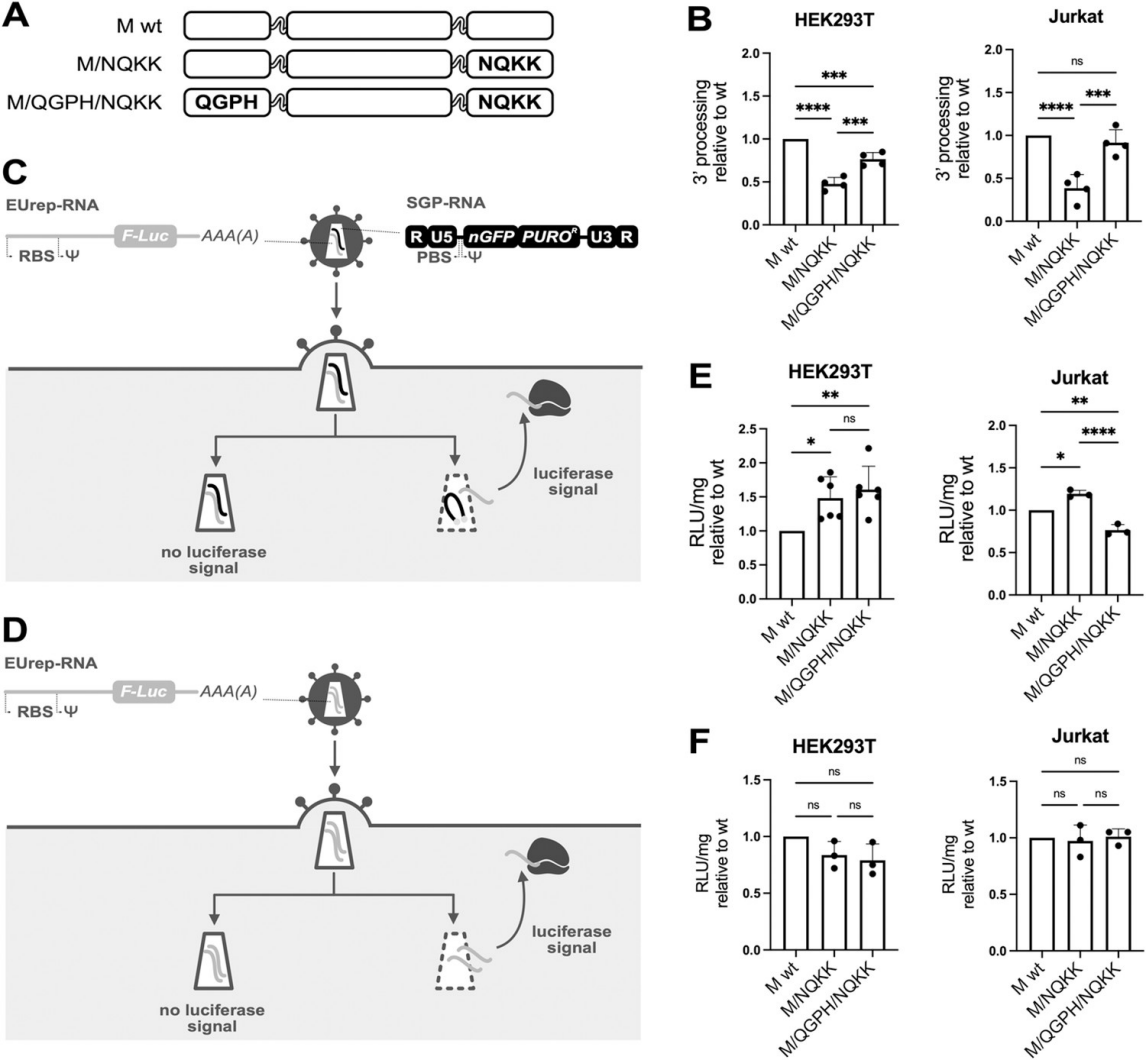
The fate of the reverse transcription products in the presence of the NOG motif. (**A**) Schematic representation of the IN used to evaluate the effect of the NOG motif on 3’ processing. When mutated with respect to the sequence of the wt, the amino acids of the NOG or of the CLA motifs are shown in capital letters. (**B**) Efficiency of 3’ processing, relative to the wt IN, for the mutants shown in panel A, in HEK293T and in Jurkat cells (n = 4 for all samples). (**C**) Outline of the EURT assay, with reverse transcription, adapted from [35]. The two types of RNA that are co-packaged in the viral particles are shown with their essential functional features. Ψ: packaging sequence, RBS: ribosome binding site, F-luc: firefly luciferase coding sequence, AAA(A): polyA sequence, R, U5 and U3: elements of HIV-1 LTRs, PBS, HIV-1 primer binding site. The SGP-RNA also has a poly-A tale, but it is not shown for clarity, not being relevant for this experimental setting. (**D**) Outline of the EURT assay, without reverse transcription. (**E**) Luciferase expression, with reverse transcription happening inside the capsid, relative to the wt IN, for the mutants shown in panel A, in HEK293T (n = 6) and in Jurkat cells (n = 3). (**F**) Luciferase expression, without reverse transcription happening inside the capsid, relative to the wt IN, for the mutants shown in panel A, in HEK293T (n = 3) and in Jurkat cells (n = 3). Data are shown as average ± SD. ****p ≤ 0.0001. ***p ≤ 0.001. **p ≤ 0.01. *p ≤ 0.05. ns, not significant (one-way ANOVA with Tukey’s multiple comparisons correction).

We have shown that replacing the NKNK sequence in the CLA motif of IN M by the sequence NQKK decreases the amount of reverse transcription products by a two-fold factor (Fig 3E). To characterize further the steps of the infectious cycle that are affected by this replacement, we measured the efficiency of 3’ processing of this mutant that resulted to be half that of the wt enzyme (Fig 6A and 6B). Interestingly, inserting the sequence of the NOG motif in this mutant (Fig 6A), rescued the defect, partially in HEK293T, and totally in Jurkat cells for which 3’ processing was comparable to that observed for wt IN M (Fig 6B). Combining this result to the reduction of the efficiency of reverse transcription leads to two conclusions. One is that these two defects are sufficient to explain the overall decrease in integration to 25% that of wt IN (as a result of 50% efficiency of reverse transcription combined to 50% efficiency of 3’ processing) when the NQKK sequence replaces NKNK in the CLA motif. The second is that, since the insertion of the NOG motif fully restores each of the two steps affected when NQKK is present in the CLA motif, the two motifs (NOG and CLA) should exert the same function in the infectious cycle. If this is the case, it may be expected that the two motifs, when present in the same IN should not have an additive effect and that the efficiency of integration would be the same as when only one of the motifs is present (i.e. = to wt IN). Accordingly, when we inserted the NOG motif in wt IN M (IN M/QGPH, Fig 3F), no improvement was observed for integration, nor for reverse transcription neither in HEK293T nor in Jurkat cells (Fig 3G and 3H) or for Pr55Gag processing (S5C Fig).

### Integrase mutants and stability of the viral capsid

Finally, we looked for possible differences, with the various IN mutants used, in the process of dismantling of the capsid, since this could alter the levels of RTPs available for integration, even if equal amounts of RTPs were measured in the cell. For instance, premature uncoating can lead to the dissociation of IN from the RTPs, while closed capsid prevents the RTPs from interacting with the genome of the infected cell. To this end, we used the EURT assay approach [35], in which the stability of the capsid is measured through the expression of a reporter gene carried by the VLP. The coding sequence is carried by an RNA (EUrep-RNA) that cannot be reverse transcribed but can be translated, leading to the synthesis of the firefly luciferase (Fig 6C). The experiment can also be carried out by co-packaging with the EUrep-RNA another RNA that can be reverse transcribed (in our case "SGP" RNA, Fig 6C). In this case the luciferase signal provided by heterozygous EUrep/SGP viruses will evaluate the stability of the capsid in the presence of reverse transcription, which is the condition relevant for the present study. If only EUrep-RNA is used (Fig 6D), the assay will measure the stability of the capsid in the absence of reverse transcription.

To study the stability in the presence of reverse transcription, VLPs are produced by transfection of cells that express equimolar amounts of two types of RNAs. Since the packaging and the dimerization signals are the same in the two RNAs, the resulting viral population is expected to be constituted by 50% heterozygous EUrep/SGP virions, 25% EUrep/EUrep and 25% SGP/SGP homozygous virions. While SGP/SGP viruses will not give any luciferase signal, homozygous EUrep/EUrep RNAs will interfere with the signal provided by the heterozygous EUrep/SGP particles. For this reason, the experiment was also performed in the absence of reverse transcription, to evaluate the contribution of homozygous EUrep/EUrep virions to the results obtained in the presence of reverse transcription and take this into account for the interpretation of the results.

The experiments were performed using wt IN M, IN M/NQKK and IN M/QGPH/NQKK (Fig 6A) in HEK293T and in Jurkat cells. In the presence of reverse transcription (Fig 6E) the replacement of NKNK by NQKK in the CLA motif led to a modest increase in the expression of the luciferase, indicating that the mutant NQKK triggers a slight decrease of the stability of the capsid. The addition of the NOG motif had no effect in HEK293T cells (Fig 6E) while it markedly increased the stability of the capsid in Jurkat cells that became even more stable than what observed with wt IN M (Fig 6E). In the absence of reverse transcription, instead, no change in the stability of the capsid was observed among the different mutants and cells tested (Fig 6F). Therefore, the specific changes in the stability of the capsid observed in the experiment performed in the presence of reverse transcription are due to the heterozygous virions and are thus related to the ongoing reverse transcription in the viral particles.

## Discussion

In this work, we document that integrases of HIV-1 groups M and O have developed two phylogenetic-group specific functional motifs that can cross-complement each other. One motif (CLA) is located in the CTD of the protein of HIV-1 group M, the other (NOG) in the NTD of isolates of HIV-1 group O. This observation highlights that, depending on the phylogenetic sequence considered, two different domains of the same HIV-1 protein carry out functions that can mutually complement each other during the infectious cycle.

We previously showed that, when at least two K are present among the four amino acids that constitute the CLA motif, the positions of the individual residues can be permutated without affecting integration in eight of the ten possible combinations [30]. In the two other cases, integration was significantly reduced with the most marked decrease (to around 25% of the wt IN M) observed with the sequence N_222_Q_240_K_254_K_273_. This phenotype was confirmed in this study in HEK293T cells and, for the first time, shown also in Jurkat cells (Figs 3E and 6B). Despite this, NQKK is highly conserved in HIV-1 O, raising the question of how it could have been selected. We find here that HIV-1 IN O has a motif in its NTD (NOG, Q_7_G_24_P_41_H_44_) that allows to bypass the need for the CLA motif, ultimately yielding levels of integration comparable to IN M. Indeed, when the NOG motif is inserted in an IN M where the CLA sequence has been mutated into NQKK, the levels of integration are brought back to those of wt IN M. This is achieved by compensating the defaults in the same specific steps (reverse transcription and 3’ processing) that were caused by the NQKK sequence, suggesting that the two motifs exert the same, or at least very similar, functions.

In Jurkat cells these effects were concomitant to an increase of the stability of the capsid. Discrepancies in the uncoating kinetics/pathways dependent on the cell type were previously reported [36–39]. This is probably due to cell-specific determinants involved in the capsid stability, as it could be the different effects observed for host factors known to interact with the capsid in different cells [40–44]. Therefore, the fact that we observed a different phenotype in HEK293T and Jurat cells for capsid stability could be explained by a cell-specific effect on this uncoating step. Dismantling of the viral capsid is a central step in the control of infectivity [45]. An implication of the IN in ensuring the optimal stability of the viral core by favoring the interaction between the capsid protein and cyclophilin A had been previously described [46]. Reverse transcription favors dismantling of the capsid core *in vitro* and the generation of full-length RTPs has been proposed to be the main motor promoting its disassembly [47,48]. The longer permanence of the reverse transcription complex in the core that is observed in Jurkat cells, might account for the higher increase in 3’ processing for this cell type (Fig 6B). Indeed, since 3’ processing occurs right after reverse transcription, when a longer time is allotted to this process before the capsid is dismantled, it could benefit from a confined environment that keeps a high concentration of the components of the reaction, as it was previously observed for reverse transcription [49–51].

The results concerning the stability of the capsid rule out the possibility that our mutants could be impaired in the packaging of gRNA. Indeed, the CTD of IN binds the viral RNA and altering this interaction results in dislocation of the gRNA outside the capsid, severely affecting reverse transcription [24,26]. The fact that the alteration of the stability of the capsid we observe is found exclusively for the viruses where reverse transcription was allowed (Fig 6B–6F) indicates that the dislocation of the gRNA is not at the origin of this result. This observation does not exclude the possibility that our results reflect an altered interaction of IN with the gRNA, but only that altering this interaction does not cause the default discussed above.

**Fig 7:**
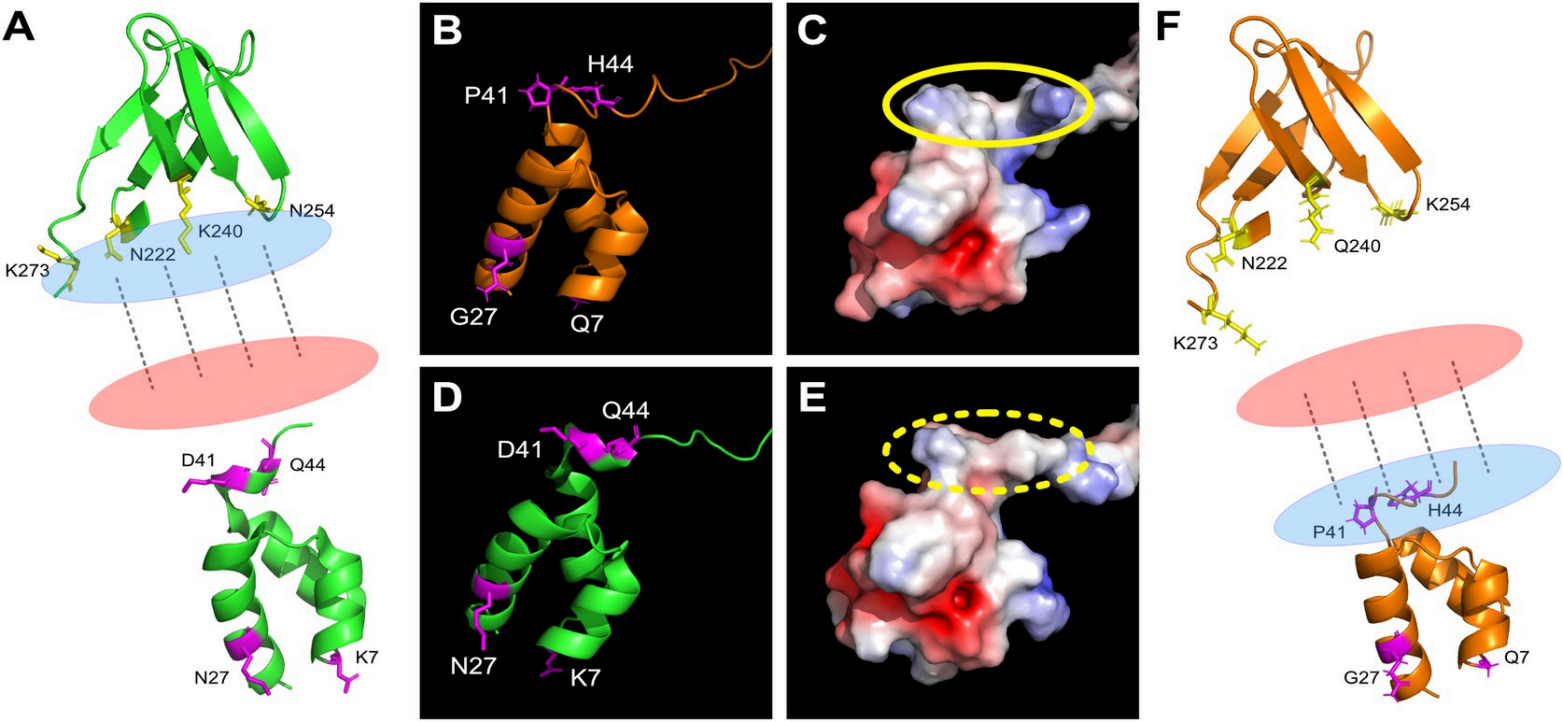
Model for the complementation of the CLA and NOG motifs. (**A**) CTD (220–274) and NTD (1–47) of HIV-1 M IN, PDB 6PUT [21], are shown in green, facing each other. This distribution could happen between two different IN being part of the same intasome. In this case, in the CTD, the CLA motif (in yellow) is forming a positively charged surface (in blue) that is interacting with a negatively charged partner (in red). In magenta are shown the amino acids occupying the NOG positions. (**B**) HIV-1 O NTD (1–47, in orange) with the NOG motif highlighted in magenta. The structure of HIV-1 O NTD was obtained with AlphaFold2. (**C**) Surface electrostatic potential of HIV-1 O NTD (panel B). The yellow circle shows the positive surface exposed by the NOG motif. (**D**) HIV-1 M NTD (1–47, in green) with the amino acids occupying the NOG positions highlighted in magenta. (**E**) Surface electrostatic potential of HIV-1 M NTD (panel D). The yellow dashes are showing the lack of the positive surface found in HIV-1 O NTD. (**F**) HIV-1 IN O CTD (220–274) and NTD (1–47), both obtained with AlphaFold2, are shown in orange, facing each other. Here, the positive surface (in blue) is formed thanks to the presence in the NTD of HIV-1 O IN of the NOG motif (in magenta), interacting with a negatively charged partner (in red). In yellow are shown the amino acids occupying the CLA positions in HIV-1 O IN CTD.

How could two different domains have converged to ensure such similar (if not the same) functions? The simplest explanation is that they interact with the same molecule. We showed that the three first residues of the motif (N_222_K_240_N_254_) form a positively charged surface, absent in the case of the N_222_Q_240_K_254_ sequence [30]. This surface was proposed to interact, possibly with the contribution of the additional K_273_, with a repetitive, negatively charged partner [30] (Fig 7A) as the backbone of DNA or RNA molecules. In HIV-1 O IN, the presence of the NOG motif is predicted to induce, particularly due to P41 and H44, the formation of an alternative positively charged surface, absent in HIV-1 M IN-NTD (Fig 7D and 7E), which could drive the interaction to involve preferentially the NTD (Fig 7F).

A possible scenario for the emergence of these two alternative motifs is that the NOG motif was established in the simian virus infecting gorillas (either by fixation of an inherited SIVcpz*Ptt* sequence or by emergence and subsequent fixation of the QGPH sequence) where it is highly conserved, at least within the limits of an analysis carried out on only 8 sequences available for SIVgor. The emergence of the CLA motif, instead, appears to date after transmission of the virus to the human host, since no conservation is found in this region in SIVcpz*Ptt*, although an overall trend for the presence of amidic and basic residues (as those composing the consensus sequence of the motif in HIV-1 M) is found. The fact that none of the combinations of these amino acids was selected in the simian virus, which instead occurred after transfer to humans, suggests that, in simian cells, the function exerted by this motif was either not required or not so important as it is in humans, allowing the co-existence of multiple functional sequences. Another possible explanation for the sequence diversity observed at SIVcpz*Ptt* CLA positions is that these amino acids were involved in a dynamic co-evolution process with a rapidly evolving molecular interface of, for example, another protein. In both cases it is tempting to speculate that the emergence of the NKNK motif was part of the process of adaptation to the new host.

When we inserted the NKNK motif in the RT-IN coding region of the SIVcpz*Ptt* MB897 strain and generated a chimeric HIV-1 carrying RT-IN of SIVcpz*Ptt*, integration was around 10% of that observed with its wt sequence (KKKK) in Jurkat cells (Fig 5D). Altogether, these results rather support the view that, once the simian virus has been transferred to humans, both sequences (RT and IN) have undergone a stepwise adaptation process to the new host that finally generated the genetic context in which the NKNK sequence in the CLA positions became optimal. The genetic flexibility that we described for the CLA locus, with several permutated sequences retaining integration ability [30], could constitute what remains of the swarm of sequences generated by genetic drift and from which selection for the successful NKNK sequence occurred.

Dominant epistasis, relieving selective pressure from the CLA motif, would have allowed this region of IN O to develop, potentially, new accessory functions. Indeed, HIV-1 integrase is a multifunctional protein that, logically, acquired its diverse functions, and optimized those already acquired, progressively during evolution. Increasing evidence supports the notion that in multifunctional proteins, the initial steps toward the establishment of a new function are undertaken by genetic drift before selection for the new function is applied [52–54]. Intra-patient expanding HIV populations are characterized by extensive genetic drift, driven by neutral selection [55], thereby creating favorable conditions for the generation of new functionalities in its proteins [56]. The presence in the CLA positions of SIVcpz*Ptt* of the same type of amino acids that would have then generated the NKNK motif in HIV-1 M, but still without selection for a specific sequence, could constitute a snapshot of such early phases of genetic drift in the process of generation of what will then become an essential motif for integration in HIV-1 M.

In conclusion, this work sheds light on crucial aspects of the emergence of two phylogenetic-group specific motifs of the integrase of HIV-1, from their simian ancestors across the barrier of the zoonotic transmission to humans. By deciphering how optimization of integration is achieved in these two cases, this work contributes to improve our understanding of the rules governing viral evolution and their role in the zoonotic transmissions.

## Methods

### Cell lines

HEK293T and Jurkat cells were obtained from the American Type Culture Collection (ATCC). HEK293T were cultured in DMEM while Jurkat were cultured in RPMI. Both mediums were completed with 10% fetal bovine serum and 1% PenStrep. HEK293T and Jurkat culture conditions were at 37°C in 5% CO_2_.

### Viral strains and sequence alignments

The primary HIV-1 isolates used in this study were: isolate HXB2 (GenBank accession number: K03455.1), isolate A2 (GenBank accession number: AF286237) from group M, subtype A2, (named "isolate M" in this study) obtained from the NIH AIDS Research and Reference Reagent Program; isolate RBF206 (GenBank accession number: KU168298) and isolate BCF120 (GenBank accession number: KU168297) both from group O, kindly provided by J.C. Plantier (CHU Rouen, France). Isolates AF286237 and RBF206 were chosen because they were used in the work that originated the present study [30]. Isolate BCF120 was chosen as the isolate O carrying the same sequences as the consensus ones in the two motifs considered in this work. The SIVcpz*Ptt* isolate employed in this work is the MB897 (GenBank accession number: EF535994) and it was chosen being one of the two isolates which are the most phylogenetically related to group M.

For the creation of the conservation logos, by using WebLogo (http://weblogo.threeplusone.com) [57,58], we performed sequence alignments using the QIAGEN CLC Genomic Workbench 22 that employs the progressive alignments approach [59]. All the sequences were obtained from the Los Alamos National Laboratory HIV database (https://www.hiv.lanl.gov/content/index).

### Plasmids and molecular cloning

The plasmid p8.91-MB previously described [30], was used as backbone for all cloning procedures. Therefore, all our constructs have the *gag* and the protease-coding sequences from HXB2 (HIV-1 group M). RT and IN coding-sequences, instead, varied. In isolate M the RT and IN was from isolate A2, while in isolates HIV-1 O it was either from isolate O206 or O120. In the chimpanzee isolate the RT and IN was from SIVcpz*Ptt* MB897. All IN mutant coding sequences were inserted between the *BspE*I and *Sal*I restriction sites of p8.91-MB by Gibson assembly. The plasmid used to produce the genomic RNA of the VLPs, carrying the two reporter genes used to evaluate integration efficiency (nGFP and PURO^R^), is a modified version of the previously-described pSRP [30] where the nuclear RFP was replaced by the nuclear GFP, giving the pSGP.

The pEUrep-RNA [35] was kindly provided by Andrea Cimarelli. The plasmid is coding for an mRNA containing the RNA packaging sequence (Ψ) and the cDNA of the Firefly luciferase followed by a polyA signal.

Two plasmids, both previously described [30], were employed for the creation of standard curves in the quantitative PCR assays. The pJet-1LTR for the detection of late RTPs and the pGenuine2LTR for the evaluation of the 3’ processing efficiency.

### Transfection and VLPs collection

To produce virus-like particles (VLPs) HEK293T cells were co-transfected with the plasmid coding for the vesicular stomatitis virus glycoprotein (VSV-G) [60], the plasmid carrying HIV-1 Gag-Pol gene (p8.91MB with different IN) and the plasmid with the modified viral genome with the reporter genes to follow the infection (pSGP). For the EURT assay the pEU-repRNA plasmid, coding for the EU-repRNA, was either added to the mix or used in the place of pSGP. All transfections were done by using 5 μg of total DNA and polyethyleneimine (PEI, Polyscience) following the manufacturer’s instructions. The medium was changed after 6 h and VLPs were collected and filtered with a 0.45 μm filter after 48–72 hours. The amount of VLPs was estimated by quantifying the p24 via ELISA (Fujirebio).

### Western blot analyses

The same volume of VLPs was concentrated by centrifuging them through a 20% sucrose for 2 h at 20,000 g and at 4°C. Pellets were resuspended in 3x Laemmli buffer and viral proteins were separated on a Criterion TGX Strain-Free 4–15% gradient gel (Bio-Rad) and then blotted on a PVDF membrane. To evaluate Pr55Gag proteolytic processing, polyproteins and mature capsid proteins were detected by probing the membrane with a mouse monoclonal anti-CA primary antibody (NIH AIDS Reagent Program) and a secondary anti-mouse HRP-conjugated antibody (Millipore). ECL reagent (Bio-Rad) was added to the membrane and images were taken with Bio-Rad Chemidoc Touch and analyzed with the Image Lab software (Bio-Rad). The Pr55Gag processing efficiency was expressed as the ratio of mature CA signal on the total CA signal (unprocessed, partially processed and fully processed CA proteins).

### Quantitative PCR for viral DNA and its forms

HEK293T or Jurkat cells were transduced by spinoculation with polybrene (Sigma-Aldrich) and an amount of VLPs corresponding to a nominal MOI of 1. Prior to infection, VLPs were incubated with Benzonase nuclease (Sigma-Aldrich) to remove non-internalized DNA. 24-hours post-transduction (hpt) cells were collected, and total DNA was extracted with DNeasy Blood & Tissue Kit (QIAGEN). All qPCR assays were designed with the Taqman hydrolysis probe technology using the IDT Primers and Probes design software (IDT), with dual quencher probes (one internal ZEN quencher and one 3’ Iowa Black FQ quencher). qPCRs were performed with the iTaq Universal Probes Supermix (Bio-Rad) on a CFX96 (Bio-Rad) thermal cycler according to the manufacturer’s protocols. Standard curves and analysis were conducted with the CFX Manager (Bio-Rad).

Late reverse transcription products were quantified with oligos amplifying the U5-Psi junction. This was normalized by the amount of genomic DNA that was quantified by amplifying an exon of the actin gene. Absolute quantification was performed by creating a standard curve with known quantities of pJet-1LTR for RTPs and the genome extracted from a known quantity of cells for actin quantification.

To evaluate the 3’ processing efficiency we first quantified the quantity of 2LTR circles (2LTRc) with oligos and probe annealing to the 2LTRc junction and then we evaluated the nature of this junction (perfect or imperfect, which are respectively the unprocessed and processed 3’ ends) with oligos and probes annealing specifically only to the perfect junction. The imperfect junction ratio was subsequently calculated as 1-perfect junction, where 1 represents the total amount of 2LTRc. For both 2LTRc and perfect junction quantification, the standard was prepared with pGenuine2LTR. All the oligos and probes used for the qPCR assays can be found in S1 Table.

### Evaluation of integration

HEK293T or Jurkat cells were transduced by spinoculation with polybrene and an amount of VLPs corresponding to a nominal MOI of 0.01. 24-hpt, puromycin was added to HEK293T at a final concentration of 0.6 μg/ml and integration was measured by counting the puromycin-resistant clones 1-week post-transduction. As previously shown, this method is comparable to the classical Alu-gag quantitative PCR method [30]. For Jurkat cells integration was measured by FACS 72-hpt by counting the percentage of cells expressing the nGFP. Therefore, in this analysis, we did not take into account the intensity of the signal but only the number of GFP positive cells. This time was chosen after having established that no signal would be detected using a catalytically inactive IN (D116A), to exclude the possibility that the signal of our constructions would derive from episomal forms of the viral DNA. Since integration depends on the availability of the RTPs and since reverse transcription is affected by the viral IN, in both HEK293T and Jurkat, results were normalized by the reverse transcription efficiency evaluated by qPCR (see above). Namely, the amount X_1_ of RTP was estimated for sample 1, for example. The number of puro resistant clones (P_1_) for HEK293T cells or the number of nGFP positive cells (F_1_) for Jurkat cells, was computed for the same sample. The normalized integration values were then computed as P_1_/X_1_ or F_1_/X_1_.

### Assessment of the capsid stability

As described above VLPs employed in this assay contain either two RNAs, the EUrep-RNA and the SGP-RNA, or the EUrep-RNA alone. The corresponding quantity of VLPs of a nominal MOI of 0.5 was used to transduce either HEK293T or Jurkat cells. 8-hpt cell protein extract was obtained, and Luciferase assay was performed with the Luciferase Assay System (Promega). Luminescence (Relative Luminescence Units, RLU) was normalized for protein concentration measured by the Bradford assay and therefore expressed as RLU per mg of protein extract (RLU/mg).

### Structure and molecular modelling

The NTD and CTD structures of IN M show in the manuscript belong to PDB 6PUT [21]. The NTD and CTD structures of IN O were obtained from molecular modelling of isolate O120 made with AlphaFold2 [61,62] by Patrice Gouet. The structure is available upon request. Pictures used in the manuscript were obtained with PyMOL2.5.

### Statistical analyses

All statistical tests were performed on at least three independent experiments (n is indicated in every figure legend) using Prism 9. ANOVA with Tukey’s multiple comparisons correction was used when more than three groups were compared. An unpaired t-test was used when two samples were directly compared. The numerical data used in all figures are included in S1 Data.

## Supporting information

S1 Fig Pr55Gag processing of IN tested in this work. (**A**) Results for Pr55Gag processing for the constructions shown in Fig 2A. Pr55Gag is not affected for all the constructions tested (n = 3). (**B**) Results for Pr55Gag processing for the constructions shown in Fig 2C. Pr55Gag is not affected for all the constructions tested (n = 3). (**C**) Results for Pr55Gag processing for the constructions shown in Fig 3C. Pr55Gag is not affected for all the constructions tested (n = 3). Data are shown as the average ± SD. ns, not significant (one-way ANOVA with Tukey’s multiple comparisons correction).
(TIFF)

S2 Fig HIV-1 O IN NTD O206 differs for only one substitution from the consensus sequence. The consensus amino acidic sequence of HIV-1 IN NTD O is shown above. Below, the sequence from the same region (1–46) is shown for isolate O206. Dash indicate a conserved position. Substitutions are indicated by bold letters.
(TIFF)

S3 Fig Alignment of HIV-1 group O integrases. The fifty sequences aligned were obtained from the Los Alamos National Laboratory HIV database (https://www.hiv.lanl.gov/content/index). Different colors indicate different conservation levels for each position. Alignment performed with QIAGEN CLC Genomics Workbench 22.
(TIFF)

S4 Fig Conservation in the CTD region of SIVcpz*Ptt* IN. Alignment of the CTD region (200–280) of SIVcpz*Ptt* isolates. In the first line is shown the sequence from the same region of HIV-1 M isolate HXB2. Different colors indicate different conservation levels for each position. Alignment performed with QIAGEN CLC Genomics Workbench 22.
(TIFF)

S5 Fig Pr55Gag processing of IN tested in this work. (**A**) Results for Pr55Gag processing for the constructions shown in Fig 5A. Pr55Gag is not affected for all the constructions tested (n = 3). (**B**) Results for Pr55Gag processing for the constructions shown in Fig 5C. Pr55Gag is not affected for all the constructions tested (n = 3). (**C**) Results for Pr55Gag processing for the constructions shown in Fig 3F. Pr55Gag is not affected for all the constructions tested (n = 3). Data are shown as the average ± SD. ns, not significant (one-way ANOVA with Tukey’s multiple comparisons correction).
(TIFF)

S1 Table Oligos and probes used for quantitative PCR assay. (TIFF)

S1 Data Excel spreadsheet containing, in separate sheets, the underlying numerical data presented in the manuscript. (XLSX)
