# Supplementary figures and images for: A snapshot on HIV-1 evolution through the identification of phylogenetic-specific properties of HIV-1 integrases M/O
Source: PLoS Pathog. 2023 Mar 30;19(3):e1011207. doi: 10.1371/journal.ppat.1011207 (PMC10062586; doi:10.1371/journal.ppat.1011207)

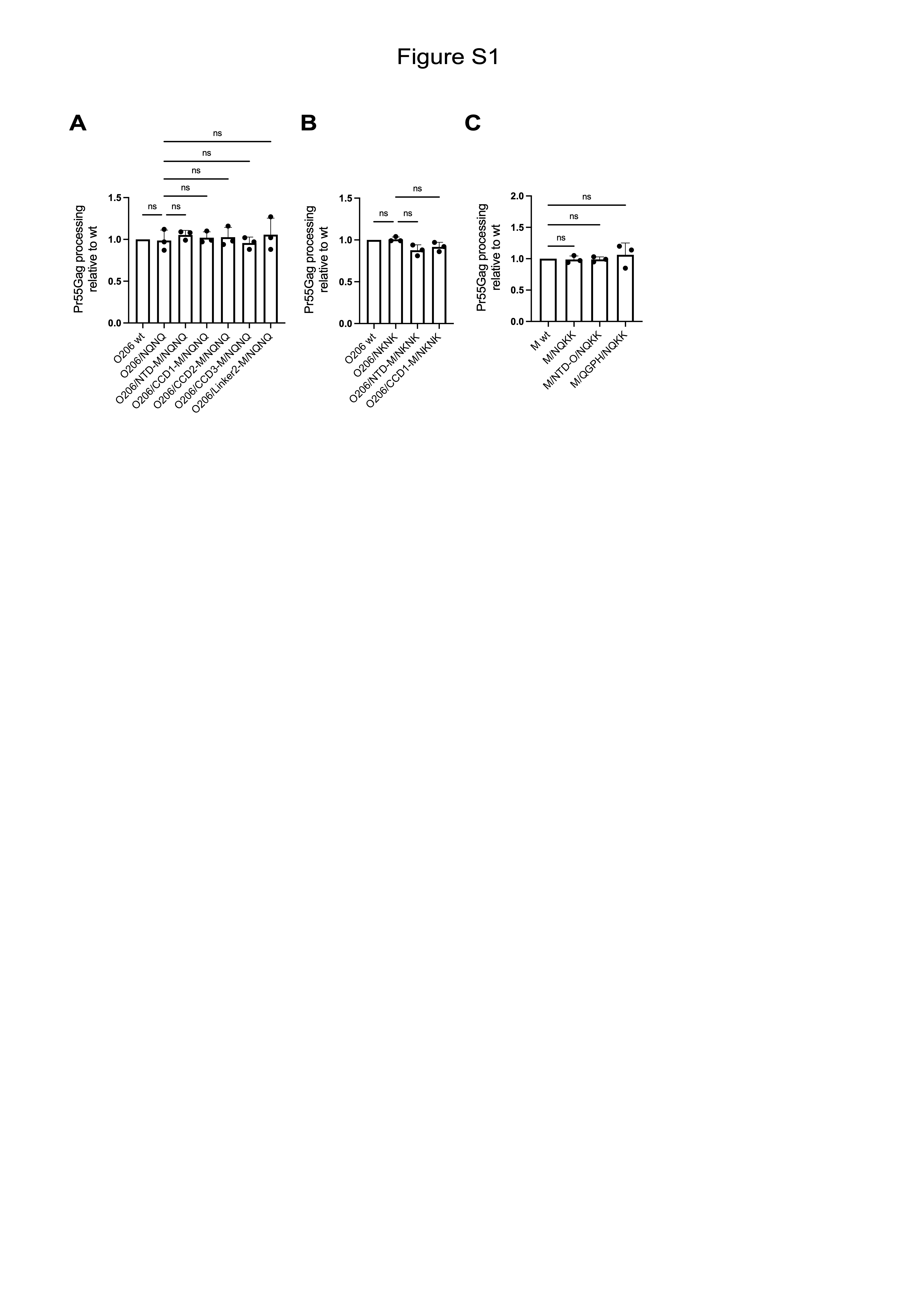

Supplement: S1 Fig — (A) Results for Pr55Gag processing for the constructions shown in Fig 2A. Pr55Gag is not affected for all the constructions tested (n = 3). (B) Results for Pr55Gag processing for the constructions shown in Fig 2C. Pr55Gag is not affected for all the constructions tested (n = 3). (C) Results for Pr55Gag processing for the constructions shown in Fig 3C. Pr55Gag is not affected for all the constructions tested (n = 3). Data are shown as the average ± SD. ns, not significant (one-way ANOVA with Tukey’s multiple comparisons correction). (TIFF) [file ppat.1011207.s001.tiff]

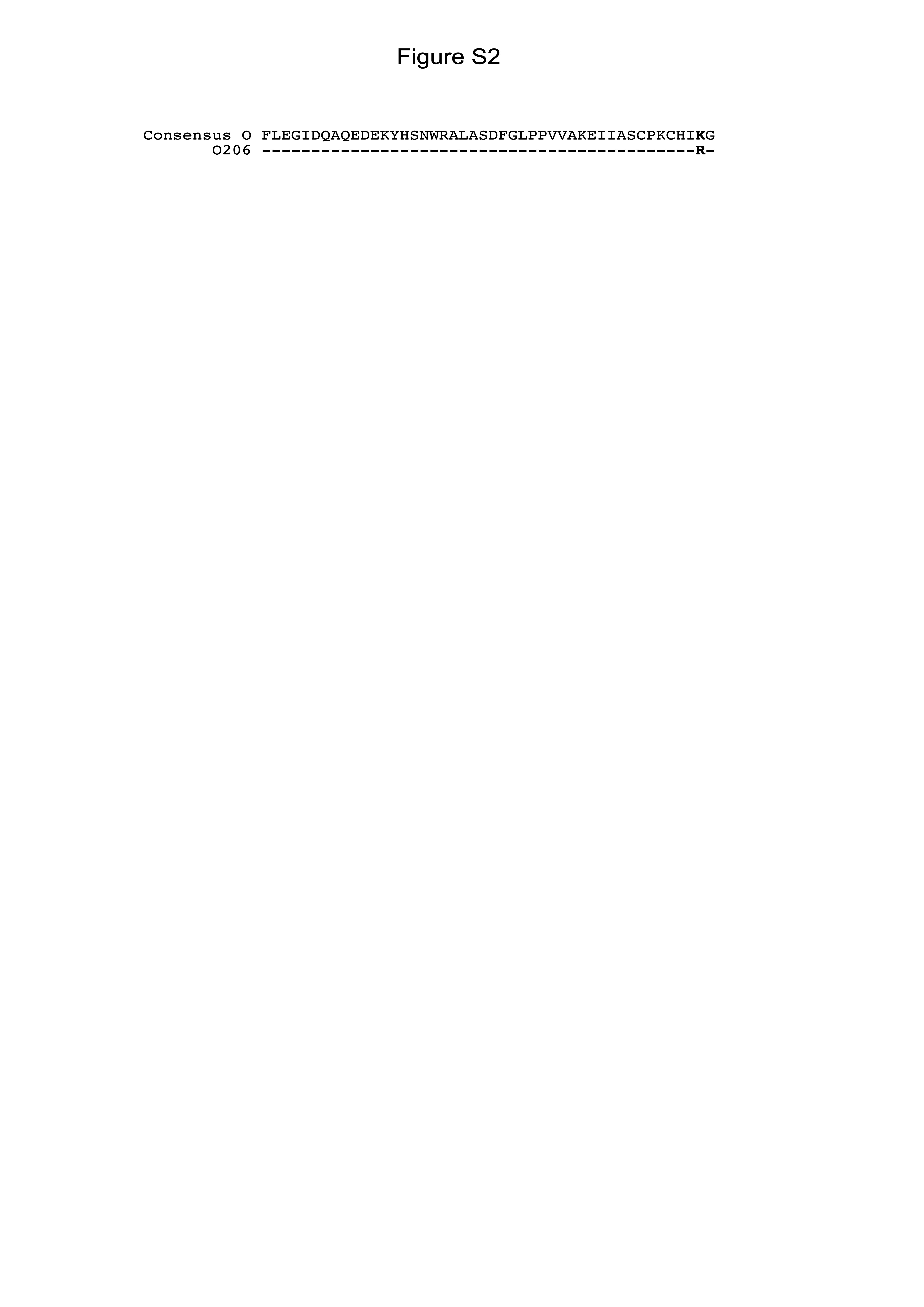

Supplement: S2 Fig — The consensus amino acidic sequence of HIV-1 IN NTD O is shown above. Below, the sequence from the same region (1–46) is shown for isolate O206. Dash indicate a conserved position. Substitutions are indicated by bold letters. (TIFF) [file ppat.1011207.s002.tiff]

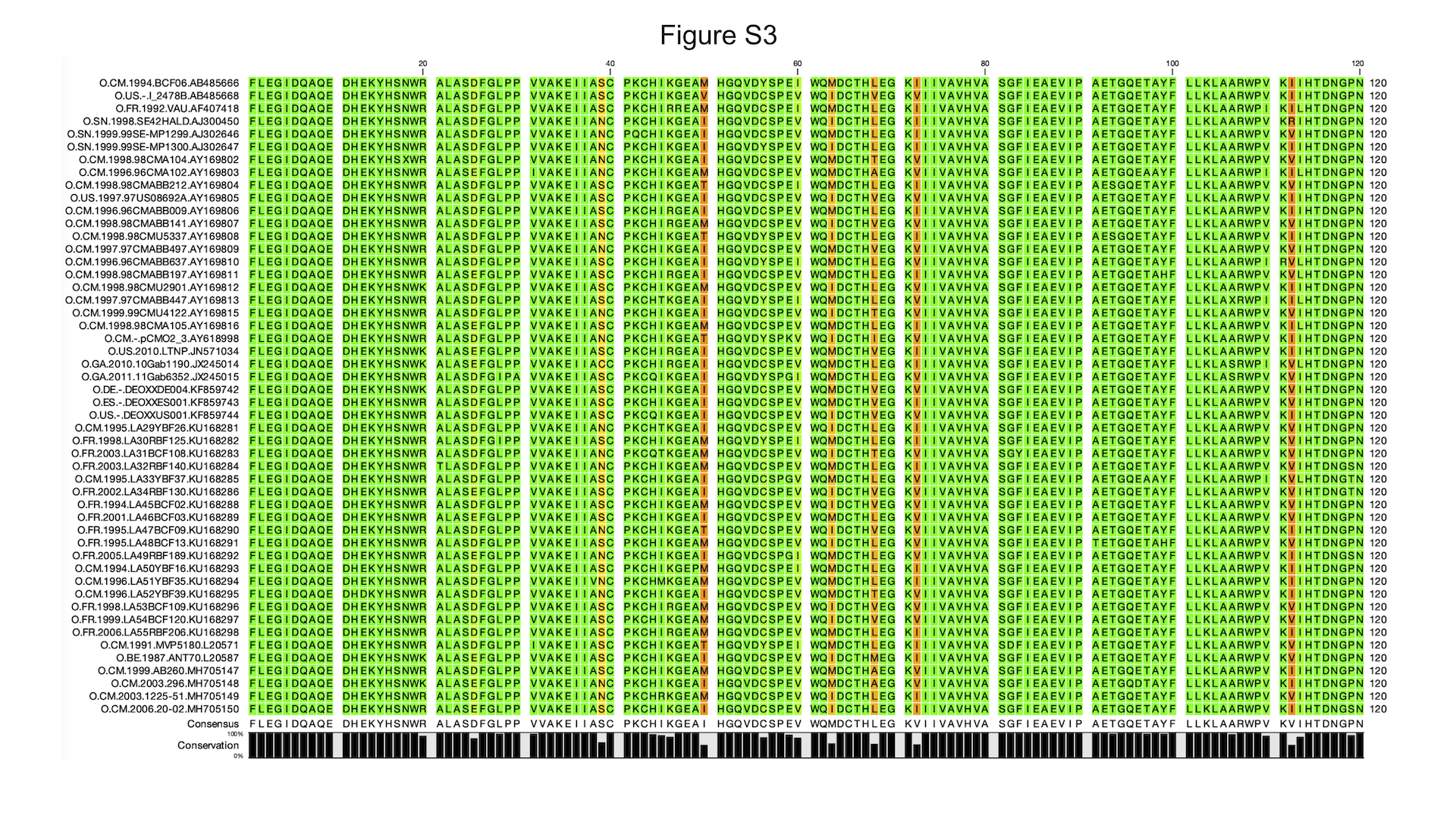

Supplement: S3 Fig — The fifty sequences aligned were obtained from the Los Alamos National Laboratory HIV database (https://www.hiv.lanl.gov/content/index). Different colors indicate different conservation levels for each position. Alignment performed with QIAGEN CLC Genomics Workbench 22. (TIFF) [file ppat.1011207.s003.tiff]

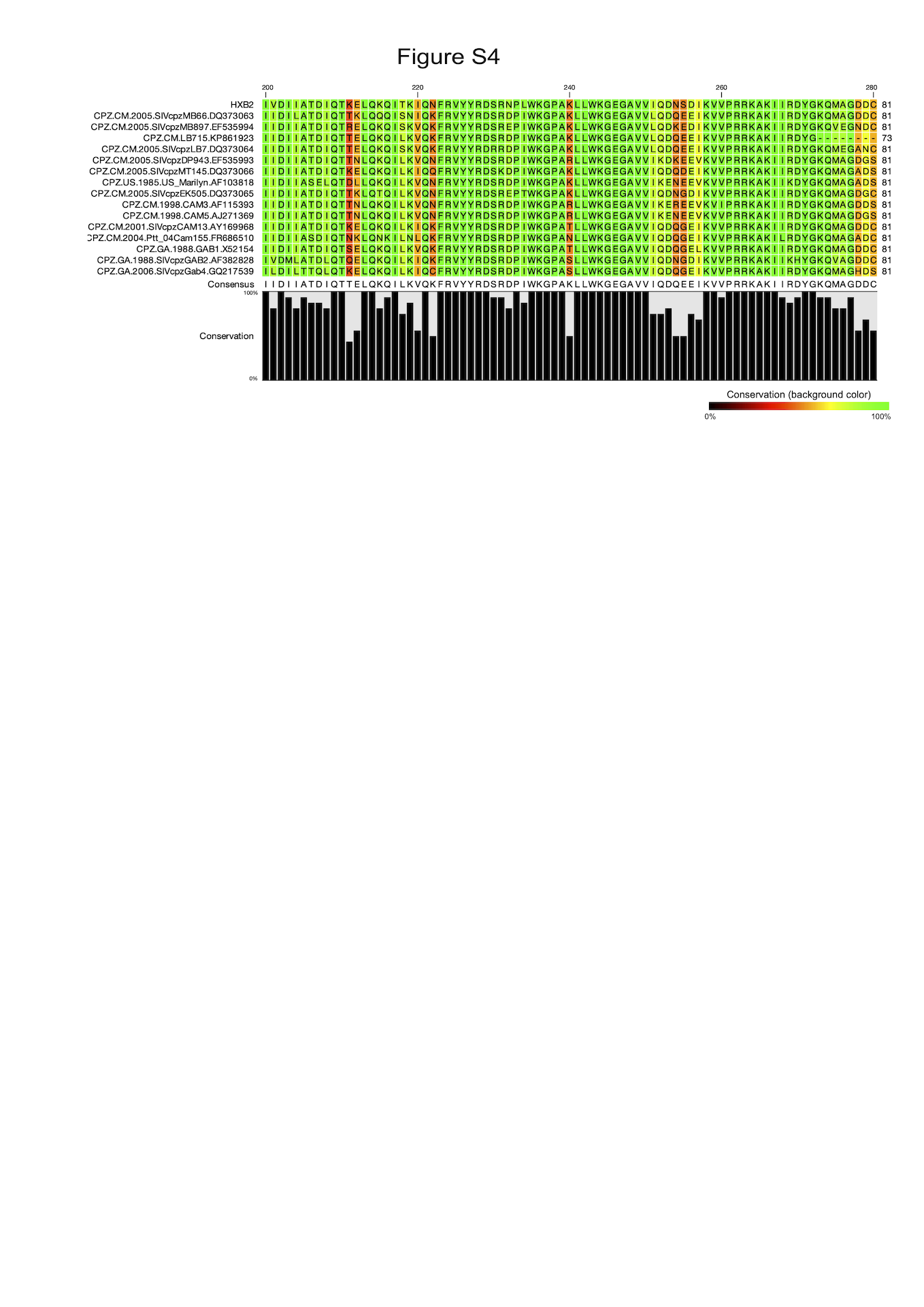

Supplement: S4 Fig — Alignment of the CTD region (200–280) of SIVcpzPtt isolates. In the first line is shown the sequence from the same region of HIV-1 M isolate HXB2. Different colors indicate different conservation levels for each position. Alignment performed with QIAGEN CLC Genomics Workbench 22. (TIFF) [file ppat.1011207.s004.tiff]

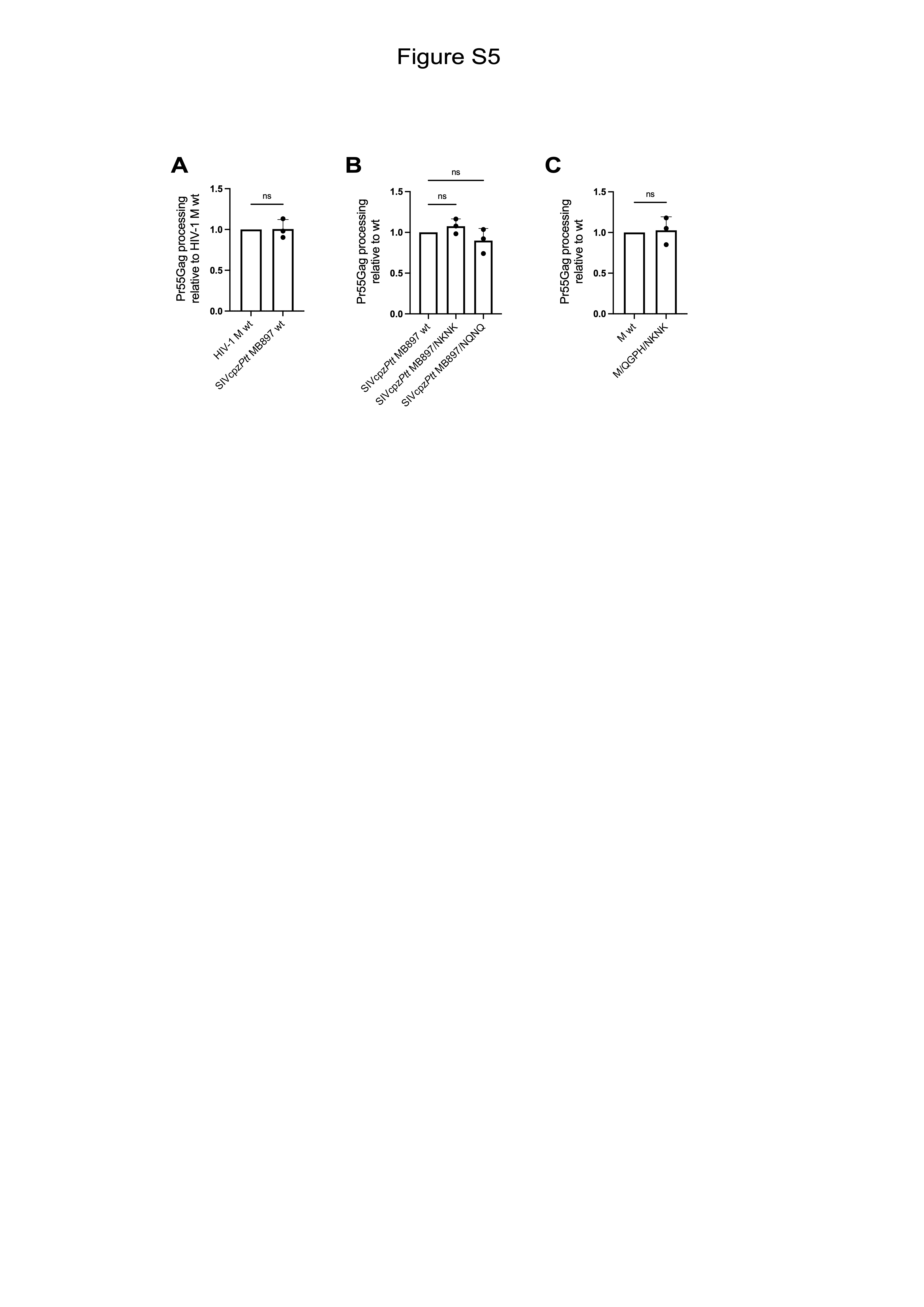

Supplement: S5 Fig — (A) Results for Pr55Gag processing for the constructions shown in Fig 5A. Pr55Gag is not affected for all the constructions tested (n = 3). (B) Results for Pr55Gag processing for the constructions shown in Fig 5C. Pr55Gag is not affected for all the constructions tested (n = 3). (C) Results for Pr55Gag processing for the constructions shown in Fig 3F. Pr55Gag is not affected for all the constructions tested (n = 3). Data are shown as the average ± SD. ns, not significant (one-way ANOVA with Tukey’s multiple comparisons correction). (TIFF) [file ppat.1011207.s005.tiff]

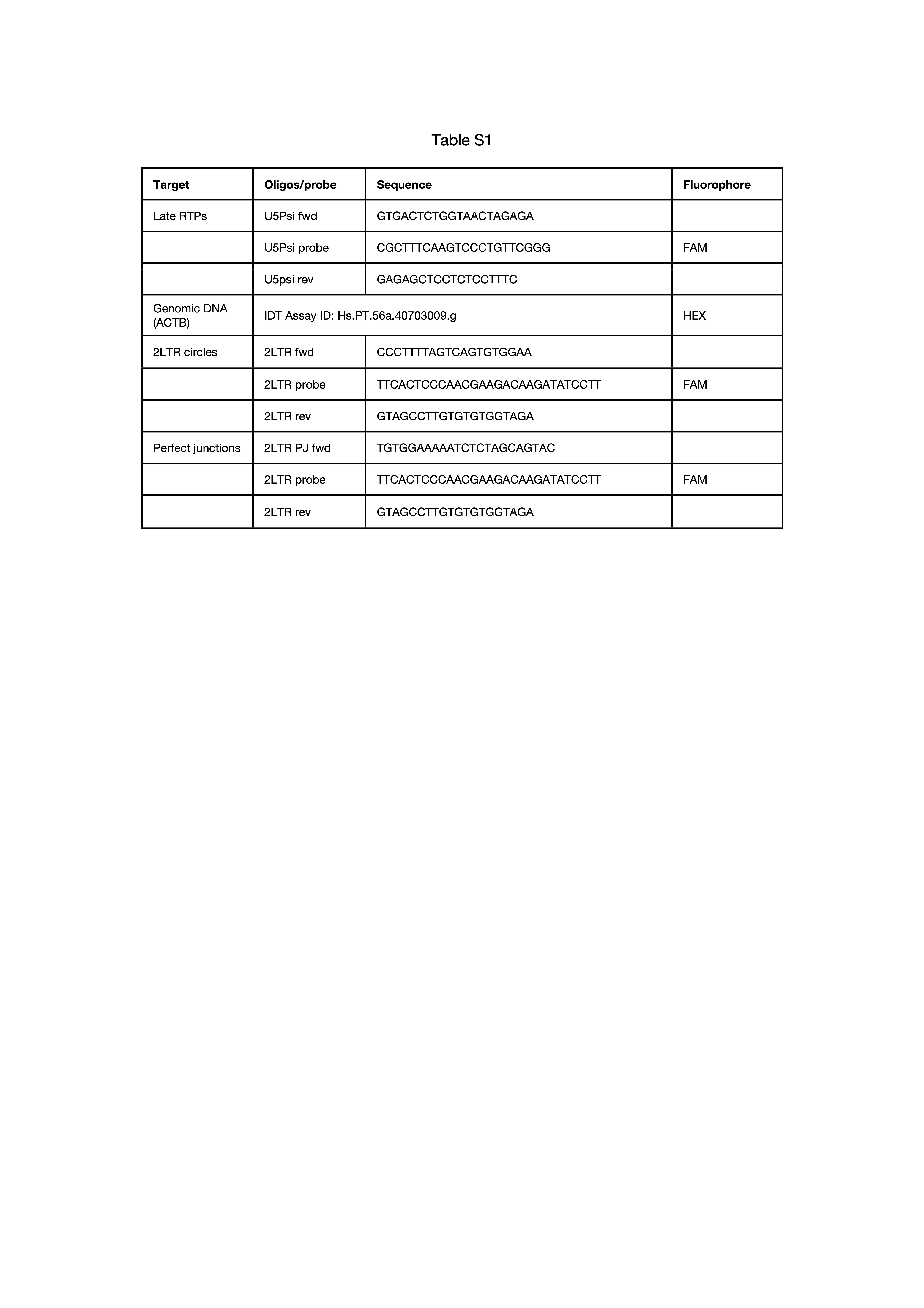

Supplement: S1 Table — (TIFF) [file ppat.1011207.s006.tiff]
